# Supplementary material for: Anti-Colitic Effects of Ethanol Extract of Persea americana Mill. through Suppression of Pro-Inflammatory Mediators via NF-κB/STAT3 Inactivation in Dextran Sulfate Sodium-Induced Colitis Mice
Source: Int J Mol Sci. 2019 Jan 5;20(1):177. doi: 10.3390/ijms20010177 (PMC6337306; doi:10.3390/ijms20010177)
Supplement: Supplementary file 1 [file ijms-20-00177-s001.pdf]

Anti-Colitic Effects of Ethanol Extract of *Persea americana* Mill. through Suppression of Pro-Inflammatory Mediators via NF-κB/STAT3 Inactivation in Dextran Sulfate Sodium-Induced Colitis Mice

Joo Young Hong, Kyung-Sook Chung, Ji-Sun Shin, Geonha Park, Young Pyo Jang and Kyung-Tae Lee

Supplementary Data

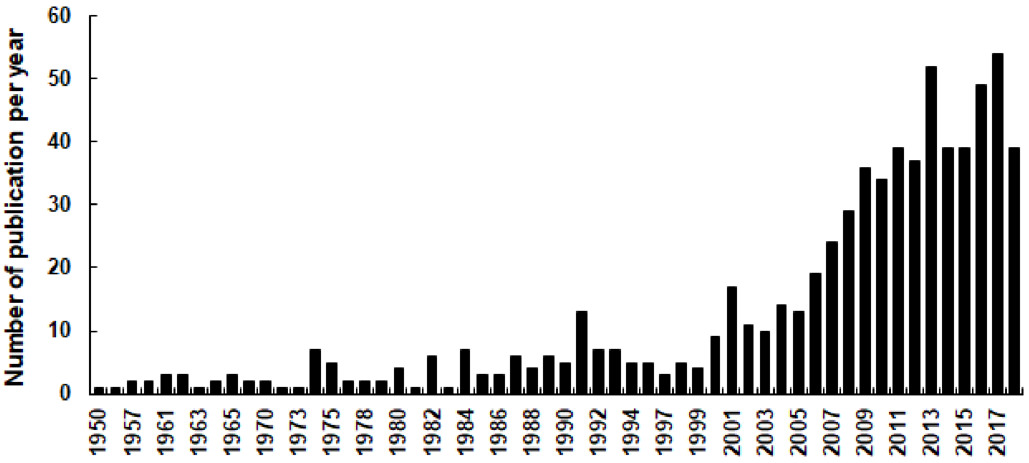

Figure S.1 The number of publication per year about *P. americana*.
